# The private versus public contribution to the biomedical literature during the COVID-19, Ebola, H1N1, and Zika public health emergencies

**DOI:** 10.1371/journal.pone.0258013

**Published:** 2021-10-22

**Authors:** Reed F. Beall, Javad Moradpour, Aidan Hollis

**Affiliations:** 1 Department of Community Health Sciences, Cumming School of Medicine and O’Brien Institute for Public Health, University of Calgary, Calgary, Canada; 2 Leslie Dan Faculty of Pharmacy, University of Toronto, Toronto, Canada; 3 Department of Economics, University of Calgary, Calgary, Canada; University of Toronto, CANADA

## Abstract

**Background:**

The private versus public contribution to developing new health knowledge and interventions is deeply contentious. Proponents of commercial innovation highlight its role in late-stage clinical trials, regulatory approval, and widespread distribution. Proponents of public innovation point out the role of public institutions in forming the foundational knowledge undergirding downstream innovation. The rapidly evolving COVID-19 situation has brought with it uniquely proactive public involvement to characterize, treat, and prevent this novel health treat. How has this affected the share of research by industry and public institutions, particularly compared to the experience of previous pandemics, Ebola, H1N1 and Zika?

**Methods:**

Using Embase, we categorized all publications for COVID-19, Ebola, H1N1 and Zika as having *any* author identified as affiliated with industry or not. We placed all disease areas on a common timeline of the number of days since the WHO had declared a Public Health Emergency of International Concern with a six-month lookback window. We plotted the number and proportion of publications over time using a smoothing function and plotted a rolling 30-day cumulative sum to illustrate the variability in publication outputs over time.

**Results:**

Industry-affiliated articles represented 2% (1,773 articles) of publications over the 14 months observed for COVID-19, 7% (278 articles) over 7.1 years observed for Ebola, 5% (350 articles) over 12.4 years observed for H1N1, and 3% (160 articles) over the 5.7 years observed for Zika. The proportion of industry-affiliated publications built steadily over the time observed, eventually plateauing around 7.5% for Ebola, 5.5% for H1H1, and 3.5% for Zika. In contrast, COVID-19’s proportion oscillated from 1.4% to above 2.7% and then declined again to 1.7%. At this point in the pandemic (i.e., 14 months since the PHEIC), the proportion of industry-affiliated articles had been higher for the other three disease areas; for example, the proportion for H1N1 was twice as high.

**Conclusions:**

While the industry-affiliated contribution to the biomedical literature for COVID is extraordinary in its absolute number, its proportional share is unprecedentedly low currently. Nevertheless, the world has witnessed one of the most remarkable mobilizations of the biomedical innovation ecosystem in history.

## Introduction

There has been extensive discussion about the contribution of the pharmaceutical industry to resolving the coronavirus pandemic, with some critics arguing that industry has profited from the support of government [1–4]. This has galvanized an ongoing and age-old debate regarding how much drug development actually costs private industry versus the public, especially considering how much is attributable to public investment in early science [5–15]. Apologists for the industry have pointed to its key role in bringing together capital and expertise to bring vaccines through clinical trials, secure regulatory approvals, and operationalize the mass manufacturing [16–18].

Within this context, numerous studies have used scientific publications to approximate the contribution of public versus private investments into the research enterprise, often finding that public contributions form the backbone of the innovation system with private industry playing a less substantial, but critical role in bringing health innovations to the marketplace [19–22]. Studies within this line of research have also included co-authorship analysis between public institutions and private firms [22]. Several investigations have used scientific bibliographic data to track the mobilization of the biomedical research enterprise during COVID-19 [23–28] with a minority of these comparing this pandemic to previous ones in order to understand the magnitude of the response [29, 30], but none have focused specifically on industry-affiliated versus non-industry-affiliated published research studies.

In this study, we use bibliographic data to examine the role of industry in the global research enterprise during public health emergencies. Our objective was to quantify the role of industry in the wide-ranging mobilization of the biomedical science community, to understand the extent to which this evolves over time, and to assess how industry’s contribution during COVID-19 compares to previous pandemics, including Ebola, H1N1 and Zika.

## Methods

We used Embase to search for publications that mentioned either COVID-19, H1N1, Zika, or Ebola (or any variant of these terms) in the abstract, extracted bibliographic data, tabulated the results, and then compared the results across the four disease areas. We consider only articles with one of the following publication types: Article, Chapter, Review, Letter, Note, and Editorial. We chose these four epidemics because each had been designated a Public Health Emergency of International Concern (PHEIC) by the World Health Organization. This led us to select COVID-19 (designated a PHEIC on 30 January 2020 [31]), Ebola (8 August 2014 [32] with a second PHEIC declared in 2019), H1N1 influenza (26 April 2009 [33]), and Zika (1 February 2016 [34]). We excluded the 2014 polio PHEIC since there were existing polio vaccines available. The disease areas of the articles were located by searching the titles and abstracts for key terms associated with or variations of names for those diseases (S1 Appendix). We used a six-month lookback window for publications prior to the PHEIC. A lookback period was used as our main objective was to closely examine across disease areas the mobilization of the scientific community following a period of high urgency, rather than the entire historical evolution prior to the PHEIC (e.g., publications for Ebola from 1976 to present day).

After extracting the publication data, the articles were categorized by reviewing the authors’ affiliations. Industry-affiliated articles were coded as those with one or more authors who listed a firm as their affiliation, such as a pharmaceutical or biotech company, rather than (or in addition to) another entity, such as a university, a governmental office, or a civil society organization. Therefore, the distinction of “industry-affiliated” includes articles that were either co-authored by one or more authors with a private affiliation or were entirely authored by authors with private affiliations. This approach was taken to establish a clear delineation rule for coding decisions that can be applied consistently across the diseases. There were a limited number of publication records in which no affiliation data were available. No affiliation data were available for a small fraction of publications. While these were not retained for our main analysis, we report on the distribution of these records across the four pandemics as well as their potential impact on our results.

Our analysis reports descriptive statistics on the number of industry-affiliated articles as well as their proportional representation of all publications examined. We plotted the number and proportion of all publications over time using a smoothing function with confidence intervals. Additionally, we plotted a 30-day publication rate (i.e., the number of articles published within 30 days of the date in question) using a cumulative sum function as well as a smooth with confidence intervals in order to more closely examine the variability in publication outputs over time. All plots were generated using the ggplot2 package in R.

## Results

Our searches within the four disease areas collectively located 126,532 relevant publications. Affiliation data was available for 120,667 (95%) and was therefore retained in our final dataset for analysis. Of these, publications for COVID-19 represented 87% (110,585) of the literature (time observed: 14 months), followed by H1N1 with 5% (6,773) (time observed: 12.4 years), Zika with 4% (5,075) (time observed: 5.7 years), and Ebola with 3% (4,099) (time observed: 7.1 years) (**Table 1**). The remaining 5% (5,856) of the publication records had no author affiliation data available. Of these 5,856 without affiliation data, the publications relevant to Ebola had the largest proportion of such records (9%, 379/4,099), followed by COVID-19 (5%, 5,119/110,585), Zika (4% 181/5,075), and H1N1 (3%, 186/6,773).

**Table 1:** Publications extracted across four pandemic areas with and without affiliation data available.

Disease area	Time observed in months (years)	Affiliation data available	No affiliation data available	Total
**COVID**	14 (1.2)	105,466 (95%)	5,119 (5%)	110,585
**Ebola**	85 (7.1)	3,720 (91%)	379 (9%)	4,099
**H1N1**	149 (12.4)	6,587 (97%)	186 (3%)	6,773
**Zika**	68 (5.7)	4,894 (96%)	181 (4%)	5,075
**Total**	316 (26.3)	120,667 (95%)	5,865 (5%)	126,532

### Industry-affiliated publications across the four pandemics

**Fig 1:**
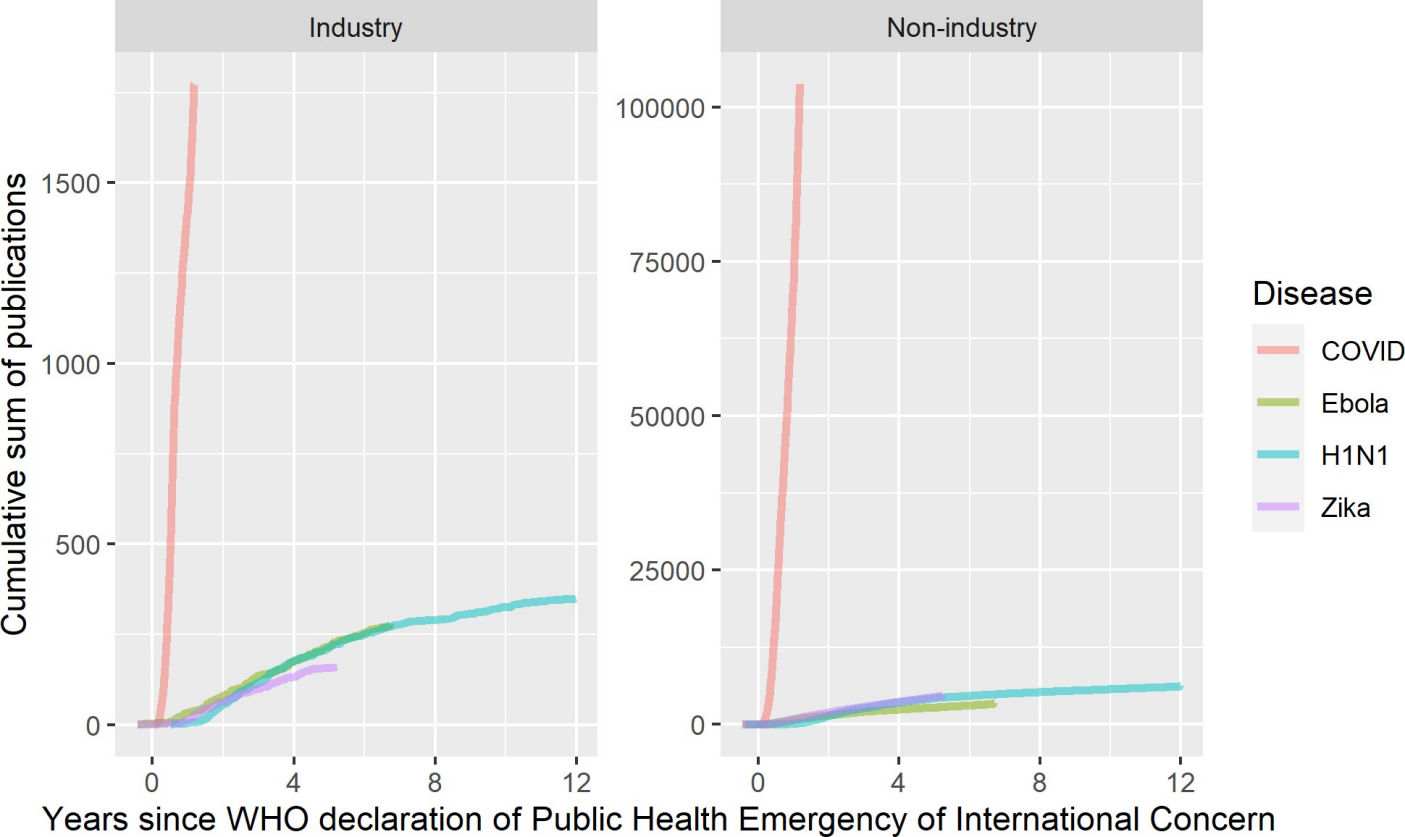
Cumulative sum of industry- versus non-industry-affiliated publications across four pandemics. Notes: The cumulative number of publications observed among Ebola, H1N1, and Zika was extremely similar for industry- and non-industry-affiliated publications alike. COVID-19 is a true outlier in reaching such a high number of publications so quickly. The absolute number is unprecedented both for industry- and non-industry-affiliated publications alike.

Of the 120,667 publications with affiliation data available, 2% (2,561 articles) had industry affiliations (**Table 2**). However, this proportion varied across the four pandemics with the highest level for Ebola at 7% (278 articles), followed by H1N1 with 5% (350 articles), Zika with 3% (160 articles), and COVID-19 with 2% (1,773 articles). At this point in the pandemic (428 days after the pandemic), the cumulative sum of publications for COVID-19 was 1,773 for industry-affiliated versus for 103,693 non-industry-affiliated publications–a number far exceeding previously observed cumulative sums after the same amount of time following the PHEIC for both industry-affiliated publications (46 times larger as compared to Ebola) versus non-industry-affiliated publications (92 times higher as compared to Zika) (Ebola: 39 versus 937; H1N1: 7 versus 271; Zika: 24 versus 1,123) (**Fig 1**).

**Table 2:** Industry and non-industry-affiliated publications across four pandemic areas.

Disease area	Time observed in months (years)	Industry	Non-industry	Total
**COVID**	14 (1.2)	1,773 (2%)	103,693 (98%)	105,466
**Ebola**	85 (7.1)	278 (7%)	3,442 (93%)	3,720
**H1N1**	149 (12.4)	350 (5%)	6,237 (95%)	6,587
**Zika**	68 (5.7)	160 (3%)	4,734 (97%)	4,894
**Total**	316 (26.3)	2,561 (2%)	118,106 (98%)	120,667

### Proportion of industry-affiliated publications over time

**Fig 2:**
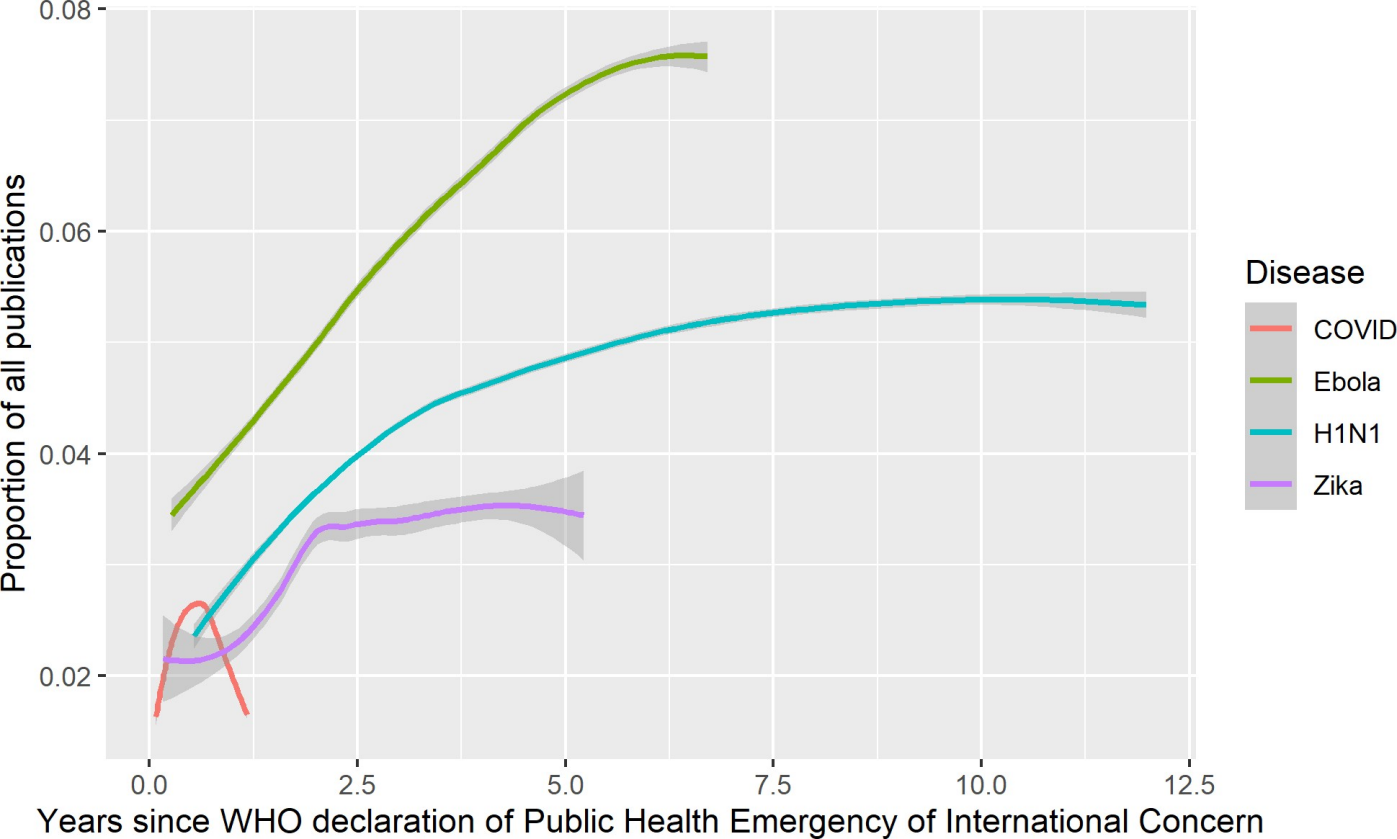
Growing proportion of industry-affiliated publications over time across four pandemics. Notes: The proportion of industry-affiliated publications varies by disease area and has generally appeared to build or stabilized over time, with large exception to COVID-19 more recently which rose above 2.5% before dropped to 1% during the time observed. COVID-19 was also unique in that the proportion of industry-affiliated articles was extremely low by the end of the observation period; for example, the proportion for H1N1 is double that of COVID-19 at 14 months out from the PHEIC.

The proportion of industry-affiliated publications varied over the total time observed for each pandemic (**Fig 2**). The proportion of industry-affiliated publications built steadily over the time and eventually plateauing over the time observed for each of the three non-COVID pandemics with 7.5% for Ebola (after 7.1 years), 5.3% for H1H1 (after 12.4 years), and 3.3% for Zika (after 5.7 years). In contrast, COVID-19’s proportion oscillated from 1.4% at 4 weeks after the PHEIC, to 2.6% at 180 days after the PHEIC, and then declined back down to 1.7% at the end of the time observed (428 days). At this point in the pandemic (i.e., 428 months since the PHEIC), the proportion of industry-affiliated articles was higher for the three other diseases (Ebola: 4.0%; H1N1: 2.8%; Zika: 2.2%).

**Fig 3:**
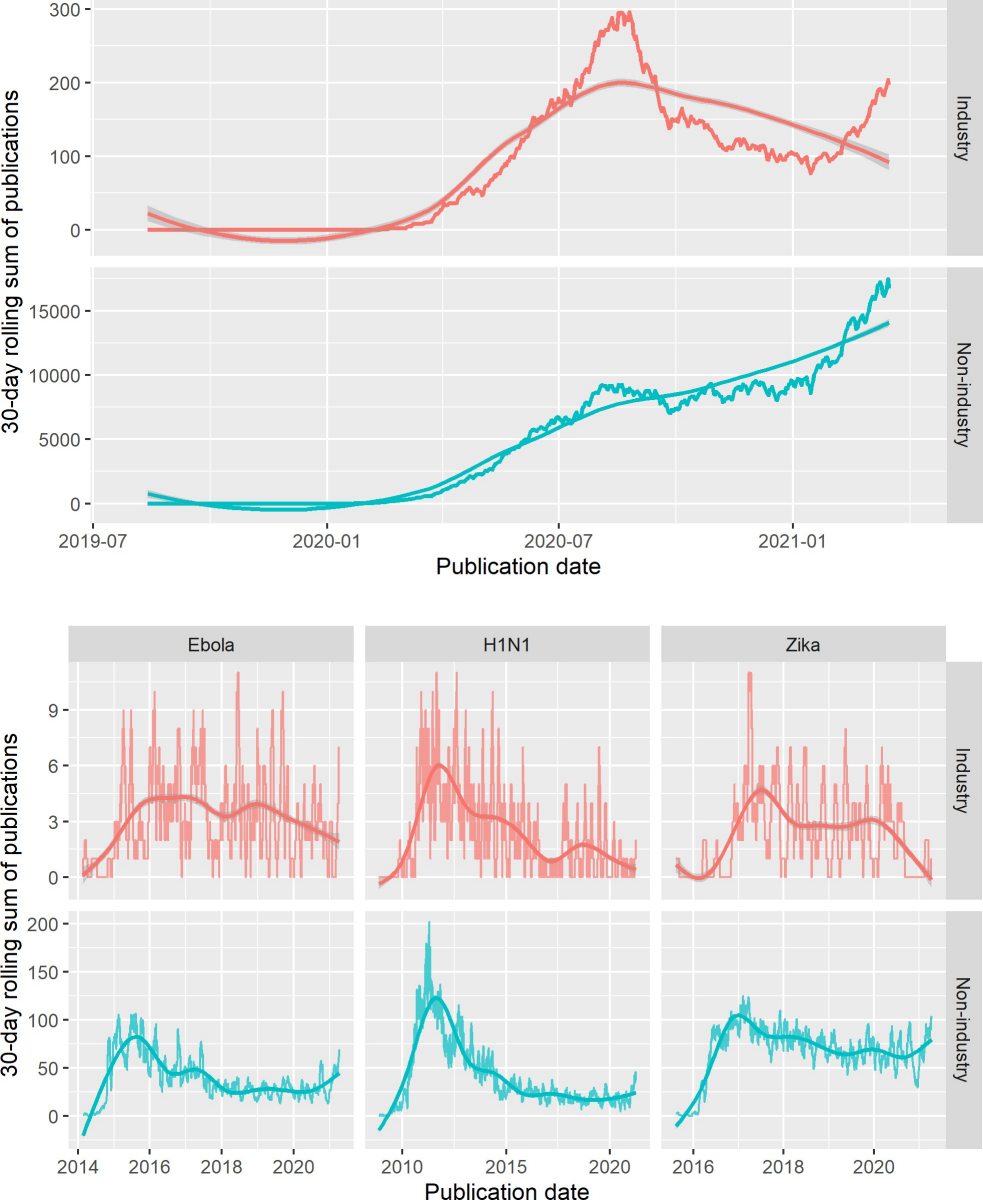
30-day publication rate of industry- and non-industry-affiliated articles across four diseases. Notes: The 30-day publication rate (i.e., a rolling sum of the total publications within 30 days of the date in question) for COVID-19 is unprecedented when compared against the publication record of Ebola, H1N1, and Zika, generally oscillated between 3–5 and 25–100 articles for industry- and non-industry-affiliates. Industry-affiliated publications appear to have peaked at nearly 300 publications per month, while non-industry-affiliated appear to continue building. This explains why the proportion of industry-affiliated publications dropped from above 2.5% of the literature back down to less than 1%. However, based on the experience of the other three disease areas, industry-affiliated publications may again accelerate in the coming months to outmatch its previous peak. Note that COVID-19 has been placed in its own panel given its uniquely high publication rate and to allow for direct comparability of the experience among the other 3 disease areas, which are on the same scale.

The proportional decline for COVID-19 occurred because industry-affiliated publications reached a peak 30-day rate of 296 publications on 26 August 2020 before declining to 77 publications on 14 January 2021. While this pace began to accelerate again in April 2021 with a last observed rate of 177 publications, non-industry-affiliated publications had been steadily climbing to a rate of 16,726 observed on that same day (**Fig 3, Panel A**). Based on the record of previous pandemics, there has been a peak in the publication rate per pandemic, but this may occur at different times for industry- and non-industry-affiliates (**Fig 3, Panel B**). It remains possible that there may be an even larger surge in industry-affiliated publications which could regain greater proportional representation in the literature in the longer term that is more commensurate with prior experience.

## Discussion

Our study has observed that industry-affiliated articles represent a small minority of the scientific publications across four recent pandemics. Industry-affiliated publications appeared more slowly but built generally over time to represent an increasing level of participation in the scientific literature, albeit still remaining below 8 percent. While it is already abundantly clear that the absolute number of industry-affiliated publications for COVID-19 is extraordinarily high, the share of industry-affiliated articles is not; in fact, it is unprecedentedly low (less than 2%) at 14 months out from the PHEIC. This result is most surprising given unprecedented amount of public funding provided to industry during COVID-19 and given the levels of proportionate engagement in the biomedical scientific literature during previous pandemics.

Similar to previous studies [23–30], we found that the mobilization of the biomedical community against COVID-19 as reflected in the record of the scientific literature is extraordinary, dwarfing what has been previously observed for other diseases. For example, by 24 May 2020 (not even 4 months after the PHEIC), one study [30] found that the number of COVID-related publications had already outnumbered the cumulative sum of publications accrued over the two years following the SARS, H5N1, MERS, Ebola, and Zika outbreaks combined.

Our findings regarding the proportion of industry-affiliated articles resonates with previous investigations, particularly in the sense that publicly funded research provides a platform from which industry can use applied research and commercialize. For example, one study [12] found NIH funding could be linked directly or indirectly to all 210 new drugs without exception that the FDA had approved between 2010–2016. Other studies have identified a link to public sector funding for 50–75% of new drugs [10, 12–13] and that as many as half of all industry patents cite prior art from public institutions [12, 14, 15].

This is not to understate the critical and substantive role of industry which has been estimated to provide two-thirds of the total investment in life sciences in the United States [35], but rather to underscore the importance of the upstream public contribution to understanding and treating disease that undergirds commercial innovation. As has been argued elsewhere [4], a possible learning from the COVID experience is that private industry responds to society’s health needs more readily when there is strong and unanimous public support; and when governments assume a proactive, substantial, and explicit role in driving the biomedical research agenda—leaving behind the market driven approach that had become status quo prior to COVID—the results are extraordinary, and more can (and should) be demanded from industry. For example, given our findings that the proportional output of publications by industry on COVID is low, there may be a need to heighten publication requirements for private industry operating with public support.

Our study is subject to certain limitations. First, while our study is as current as possible, only 14 months of data is available for COVID. There may well (and hopefully will be) a second and larger wave of industry-affiliated publications that will bring its level to be more commensurate with the experience of previous pandemics. Second, in contrast to industry, the primary responsibility of academics who largely work for public or non-profit institutions is to publish; therefore, it is unsurprising that industry publications represent a small fraction of such publications. For this reason, our study has focused upon to the proportionate experience of prior pandemics for establishing a reasonable benchmark from which to compare. Third, our investigation broadly focused upon the entire biomedical enterprise using a single, yet important repository of scientific publications. It is possible that by isolating subsets of publications—such as by intervention type (e.g., vaccines, diagnostics)—the proportion of industry-affiliated articles would be higher. However, this would be at the exclusion of the context of the broader ecosystem of knowledge and innovation which made these interventions possible in the first place. Finally, our analysis applies the most inclusive approach to industry participation: if even one author lists an industry affiliation, then the article is categorized as industry-affiliated. This may overstate the share of industry participation.

## Conclusion

The absolute number of industry-affiliated scientific articles on COVID in the biomedical literature is unprecedented when compared to the previous experience of Ebola, H1N1, and Zika. However, the proportional share of industry-affiliated articles (versus non-industry-affiliated articles) on COVID is not. In fact, as of writing, its current level is extraordinarily low at below 2 percent, perhaps because the world has seen the most stunning mobilization of the biomedical innovation ecosystem in history. A possible learning that may emerge from the COVID pandemic in the long term is an appreciation for what can be achieved with a strong role for governments in driving innovation agendas and the advantages of this strategy over a market-driven model in which private industries are left to strategically respond to lower-risk business opportunities.

## Supporting information

S1 Appendix Search terms used for Embase search. (DOCX)
