# Supplementary material for: The private versus public contribution to the biomedical literature during the COVID-19, Ebola, H1N1, and Zika public health emergencies
Source: PLoS One. 2021 Oct 22;16(10):e0258013. doi: 10.1371/journal.pone.0258013 (PMC8535173; doi:10.1371/journal.pone.0258013)
Supplement: S1 Appendix — (DOCX) [file pone.0258013.s001.docx]

**S1 Appendix. Search terms used for Embase search**

| **Disease area** | **Search terms with “main focus” of study selected in Embase’s subject headings** |
| --- | --- |
| **COVID-19** | COVID, coronavirus disease 2019, SARS-CoV-2 |
| **Ebola** | ebola hemorrhagic fever, ebola vaccine, and ebolavirus |
| **H1N1** | influenza A (H1N1), swine influenza, swine influenza virus, swine influenza vaccine |
| **Zika** | zika fever, zika virus, zika virus vaccine |
